# Kentucky Lunatic Asylum

**Published:** 1842-03

**Authors:** 


### KENTUCKY LUNATIC ASYLUM.

In the Review to which we have referred in the preceding article, Dr. Jarvis showed, from the reports sent out by most of the establishments of Europe and America, that the Kentucky Asylum has effected the smallest proportional number of cures, of the whole. This fact, together with his judicious remarks on its causes, and his suggestions for their removal, led the General Assembly to institute a full and searching inquiry into its condition, the results of which are now before us, in the Report of a Committee of its members. Most judiciously the committee recommended an appropriation for the purchase of a farm contiguous to the Asylum, on the necessity for which, and for some other improvementt, they speak in the following language.

"It is believed, that with the addition of the one hundred and seven acres, besides the incalculable advantage to the lunatics themselves, under judicious and proper management, the institution, by their labor, might be abundantly supplied with vegetables, and all the other productions of the soil.

When it is recollected that we are an agricultural people, and that a very large proportion of those who are found in the Hospital are accustomed to agricultural pursuits, and that, therefore, the cultivation of the soil will be not only useful, but agreeable to them, the calculation cannot be deemed extravagant. The Committee is fully sustained in this assumption, by the experience of the Worcester Asylum, in Massachusetts, where the lunatics have not only raised abundantly for the establishment, but have produced a surplus for sale. It is not unusual, under the judicious and enlightened administration of that institution, "to see two men ploughing in the field, quiet and attentive to their work, and performing it well; both insane, both having committed homicide, and had therefore been confined many years in prison." The pleasing spectacle is exhibited of cheerfulness and contentment. "Those who before, were a pain to themselves, and a torment to their friends, and dangerous to the public, are now mostly quiet, comfortable, and even happy and useful. The sting of their mental death is taken away."

The Committee does not rest the claims of the Asylum to this additional appropriation, on these grounds alone. They are of opinion that it is the *duty* of the Legislature to afford all the means in its power to carry out the great objects of the institution, viz: the *restoration of the insane,* and the amelioration of their unhappy lot. To imprison them, to impose upon them the unnecessary restraints of confinement, to deny to them the common use of their own limbs, to shut them out from the light of heaven, and the enjoyment of the bounties of Providence, is at war with the spirit of the age, and in opposition to the lights of experience and the discoveries of science. They should be afforded the means of every enjoyment, not inconsistent with their wretched condition.

One of the advantages which the Committee proposes to accomplish by this purchase, is, that a residence will be secured, in all respects convenient and suitable, for a Resident Physician. They do not recommend the immediate employment of such an one, but they do not hesitate to express the opinion, that if the necessary arrangements could be made, that the true interests of the institution require it. That every successful Asylum for the insane, of which the Committee has any knowledge, is afforded the services of an enlightened Physician, whose time is exclusively devoted to the investigation and treatment of mental disease, is a fact entitled to the most respectful consideration.

It is not only necessary that the Physician should understand the physical maladies with which the patient is afflicted, but he should be acquainted with his character and the causes of his mental derangement, and by a close and accurate observation, determine upon the best means for his relief. He should be enabled by his manners and character to acquire a mastery over the intellect of his patient, and to conduct it in the proper channel. It should be his province to direct the whole machinery of the institution, with this object kept steadily in view. In a word, all the means employed, not excepting the government and discipline of the establisment, should be under his immediate control. Hence the necessity of a Resident Physician, and providing a suitable and convenient place for his accommodation."

We concur in every word of this extract, and when the Report reached us, fondly hoped, that the Legislature would not adjourn without carrying out the recommendations of the Committee. That hope has, however, been disappointed; and we now call on the physicians of the State to step forth, and seek so to enlighten and animate the members of the next General Assembly, as to bring about the important ameliorations, demanded equally by the interests of humanity, and the dignity of the State. D.

